# Operation Change: A New Paradigm Addressing Behavior Change and Musculoskeletal Health Disparities

**DOI:** 10.1007/s40615-018-0473-2

**Published:** 2018-04-24

**Authors:** Lynne C. Jones, Yashika Watkins, Duanny Alva

**Affiliations:** 1 Department of Orthopaedic Surgery, The Johns Hopkins University School of Medicine, Baltimore, MD USA; 2 College of Health Sciences, Chicago State University, Chicago, IL USA; 3 Department of Medicine, Feinberg School of Medicine, Northwestern University, Chicago, IL USA

**Keywords:** Behavioral change program, Early-stage osteoarthritis, Knee pain, Obesity, Outcomes, Physical activity level

## Abstract

**Background:**

In this study, we examined the implementation and efficacy of Operation Change, a community-based, culturally sensitive program to stimulate behavioral changes in activity level and improve musculoskeletal health in African-American (AA) and Hispanic/Latina (H/L) women with obesity and early-stage osteoarthritis.

**Methods:**

Sixty-two women (32 AA and 30 H/L), 40–75 years old, with nontraumatic knee pain and body mass index values > 30, participated in a 12-week program of presentations, motivational interviewing, goal setting, and physical activities. Assessments (at 0, 6, and 12 weeks) included a demographic questionnaire, physical assessment, timed 50-ft walking test, Western Ontario and McMaster Universities Arthritis Index (WOMAC), Short Form-36 Health Survey (SF-36), 8-Item Physical Health Questionnaire (PHQ-8), and motivational interview assessment.

**Results:**

Walking time improved significantly for H/L women (*P* < 0.0001) but not AA women (*P* = 0.0759). Both groups had significant mean weight loss (*P* < 0.05) with high variability among individuals. WOMAC scores for both groups indicated decreased pain (*P* < 0.0001) and stiffness (*P* < 0.0001) and improved physical functioning (*P* < 0.0001) by 12 weeks. SF-36 results were comparable to those of the WOMAC. PHQ-8 results improved significantly for H/L women (*P* < 0.0001) but not AA women (*P* = 0.077). Participants scored the motivational interviewing component of the program favorably.

**Conclusions:**

Participation in Operation Change increased physical activity, resulting in improvements in pain and function scores. This supports a new paradigm for behavioral modification that helps AA and H/L women take an active role in living with osteoarthritis.

## Introduction

In the USA, approximately 23% of adults have self-reported, physician-diagnosed arthritis; this rate is 50% for those older than 65 years [1]. Radiographic osteoarthritis (OA) is more prevalent in non-Hispanic blacks and Mexican-Americans aged 60 years or older than in corresponding non-Hispanic whites [2]. According to the Centers for Disease Control and Prevention, a greater proportion of African-Americans (AAs) and Hispanics have activity limitations, work limitations, and severe joint pain compared with non-Hispanic whites [3]. Women make up 61% of OA patients [4], and AA and Hispanic women may have an even higher risk of developing OA [5].

The natural history of OA involves a vicious cycle of joint pain and limited mobility and is associated with comorbidities such as diabetes, fatigue, depression, and obesity. Many of these factors are interrelated, exacerbating the progression of OA. Pain may limit mobility and physical activity, leading to weight gain [6, 7]. Impaired mobility is associated with pain, instability, fear of physical activity or falling, and depression [2, 8, 9]. Obesity is associated with arthritis progression, joint pain, activity limitation, disability, reduced quality of life, need for total joint replacement, and poor clinical outcomes after total joint replacement [10, 11]. The risk of obesity is higher for women than for men [12, 13], and the association between higher BMI and depressive symptoms may be stronger for AAs than for whites [14].

Physical exercise programs for patients with early-stage OA [15] can increase strength and flexibility and reduce joint pain and chronic fatigue [16, 17]. Such several programs are available through community organizations, but few have been developed specifically for underserved minorities and/or obese women.

Operation Change is a community-based (i.e., implemented in the community using community resources), culturally sensitive (i.e., designed with knowledge of patients’ culture, acceptance of cultural differences, and adaptation of interpersonal skills to work with the community [18]) program that involves education, physical activity, group sessions, and motivational interviewing conducted by individuals of identical race or ethnicity. By incorporating these features, Operation Change is a paradigm shift from traditional exercise or weight loss programs. The goal of this exploratory study was to examine the implementation and efficacy of Operation Change to stimulate behavioral changes in activity level and improve musculoskeletal health in obese AA and Hispanic/Latina (H/L) women with early-stage osteoarthritis.

## Patients and Methods

### Study Population

Participants were recruited from the South Side of Chicago through flyers and word-of-mouth from local referral sources. The inclusion criteria were AA and H/L women, 40–75 years of age, self-reported knee pain and/or early-stage arthritis (self-reported physician diagnosis), and body mass index (BMI) of 30–45. Subjects were excluded if they reported uncontrolled medical conditions or stage-2 hypertension or were unable to stand unsupported for 10 min. Informed consent (institutional review board-approved) was obtained at the screening visit.

### Program Design

The program, conducted in 2012, took place every Saturday for 3 h over 12 weeks. The AA women met in the local Young Men’s Christian Association, and the H/L women met in a local church. Each session included 1 h each of physical activity, health-education sessions, and motivational interviewing group sessions. Light breakfast and snacks were provided. Separate individualized visits were scheduled for assessments and goal setting at baseline, 6 weeks, and 12 weeks.

#### Physical Activity

Certified health trainers and community health workers led the physical activities, including walking, low-impact aerobics, stretching, dancing, meditation, and yoga. Optional activity classes were offered twice a week. Participants were encouraged to engage in exercise during the week.

#### Education

Lectures and question-and-answer sessions were provided by allied health professionals, nurses, a nutritionist, physical trainers, and physicians. Care was taken to be culturally sensitive during selection of topics and illustrative examples. Topic selection was based on participant input and consisted of movement as medicine, living with OA, healthy eating, depression, support networks, community resources, goals for lifestyle change, and chronic diseases associated with OA and obesity. For the H/L group, guest presenters were fluent in Spanish or teamed with a translator.

#### Motivational Interviewing

Motivational interviewing addresses ambivalence to change by supporting and encouraging individuals to set goals to create change. Although many publications have addressed diet and weight loss, our focus was on increasing mobility. To achieve this, Operation Change used motivational interviewing, which is based on the highly validated trans-theoretical model (TTM) of behavioral change [19]. Application of TTM in Operation Change involved using motivational interviewing to conceptualize where participants are along the stages of change continuum to determine readiness for change and which type of “change talk” is needed to help them make a behavior change. Research reveals that behavioral change is significantly more achievable when patients are granted autonomy in setting their personal goals. The Operation Change program was concerned foremost that (1) participants set their own goals and (2) they work to meet those goals, identifying any potential barriers. Our motivational interviews were therefore focused on empowering individuals to make healthier decisions for themselves and to become more active participants in their own health.

Each participant was assigned a community-based layperson (Champion) and encouraged to share thoughts and personal experiences related to barriers to making behavioral changes. Several resources (i.e., SMART goals [20], My Steps Toward Change [21], and The Fishbone Tool for Root Cause Analysis [22]) were used to help participants identify strategies for change.

Weekly facilitated discussions examined influences that motivate positive behavior changes, overcoming obstacles, action plan development, community resources, and the importance of defining goals that are specific, measurable, attainable, realistic, and timely. Individualized weekly goals were established by each participant. Participants received one additional hour of individualized motivational interviewing by the Champions at baseline, 6 weeks, and 12 weeks (not during the Saturday program). Champions made weekly follow-up telephone calls to participants to support their participation.

### Baseline and Follow-up Assessments

Evaluations were obtained by the study coordinators at baseline, 6 weeks, and 12 weeks (Table 1). Physical assessments included measurement of weight, height, blood pressure, and resting heart rate. The Western Ontario and McMaster Universities Arthritis Index (WOMAC-LK3.0) and Short Form-36 Health Survey (SF-36 v2; OPTUM, Inc., Eden Prairie, MN) were licensed for this study. The 8-Item Patient Health Questionnaire Depression Scale (PHQ-8) was used to assess depression. Instructed to walk as quickly as possible, participants performed a timed 50-ft walking test. A motivational interviewing discussion guide evaluated participants’ perceptions of the importance of their goals, their readiness to achieve them, and their confidence in in their ability to achieve them [23, 24].

**Table 1:** Schedule of assessments during the 12-week Operation Change program

Assessment	Baseline	Program week											
		1	2	3	4	5	6	7	8	9	10	11	12
Demographic questionnaire	X												
8-Item Patient Health Questionnaire Depression Scale	X						X						X
Exercise log		X	X	X	X	X	X	X	X	X	X	X	X
Focus groups	X						X						X
Global Pain Scale	X						X						X
List of medications	X												
Motivational interviewing	X						X						X
Physical assessment^a^	X						X						X
Short Form-36 Health Survey	X						X						X
Timed 50-ft walking test	X						X						X
Western Ontario and McMaster Universities Osteoarthritis Index	X						X						X

^a^Physical assessment measured weight, height, blood pressure, and resting heart rate

### Incentives

Participants received an Operation Change T-shirt and a pedometer, bus passes as needed, and $25 gift cards for each motivational interviewing session and completed evaluation.

### Program Team

The Operation Change program team included experienced study coordinators, program assistants (Champions), and activity facilitators. The AA study coordinator was recruited by the principal investigator, and the H/L coordinator was recruited through the board of a community health workers’ organization, both located in the South Side of Chicago. Champions and activity facilitators were recruited through connections within the community, the healthcare system, and churches. Champions had to be community members or of the same race/ethnicity as the participant group. Certified health trainers and community health workers led the physical activity. H/L team members were required to have previous experience working with the H/L community and to be bilingual (Spanish/English). A faculty member from a local university instructed the Champions in motivational interviewing techniques, including using change talk and OARS (open-ended questions, affirmation, reflection, and summary statements).

### Statistical Analysis

Statistical analysis was performed using JMP, version 10, software (SAS, Cary, North Carolina) and VassarStats: Website for Statistical Computation (VassarStats.net; ©Richard Lowry, 1998–2015). Parametric data were analyzed using analysis of variance for repeated measures; the Tukey honest significant difference test was performed when the analysis of variance yielded a significant F-ratio. Pre-post testing was assessed using Student’s *t* tests for matched pairs. Frequencies were compared using likelihood-ratio chi-square tests. Significance was accepted when *P* < 0.05. In response to a request from Operation Change participants, no statistical comparisons were made between the AA and H/L groups.

## Results

### Study Population

**Fig. 1:**
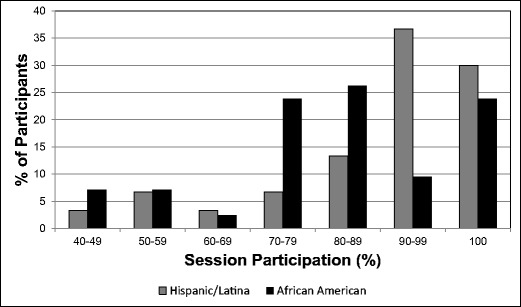
Rate of participation of African-American and Hispanic/Latina women in Operation Change. Session participation is the percentage of sessions that were attended by each participant

Ninety-two women (54 AA and 38 H/L) were screened. Twenty-two failed the screening (16 AA and 6 H/L), and 8 dropped out of the study (6 AA and 2 H/L), 1 of whom dropped out after completing 6 weeks of the program. Consequently, 62 participants (32 AA and 30 H/L) completed the 12-week program. As indicated in Table 2, mean ages were 61 years (range 41–75 years) for AA women and 52 years (range 45–66 years) for H/L women. Mean baseline BMI values were 36.6 ± 8.6 for AA women and 35.6 ± 5.6 for H/L women. Regarding weekly participation, 83% of AA and 87% of H/L women attended at least 70% of the sessions during the 12-week program (Fig. 1).

**Table 2:** Demographic characteristics of 62 women participating in Operation Change

Group	No. of participants	Mean (range) age, years	Mean ± SD BMI
African-American	32	61 (41–75)	36.6 ± 8.6
Hispanic/Latina	30	52 (45–66)	35.6 ± 5.6
Total	62	57 (41–75)	35.4 ± 5.0
*P* value	NA	< 0.0001	0.6198

*BMI* body mass index, *NA* not applicable, *SD* standard deviation

### General Health

**Fig. 2:**
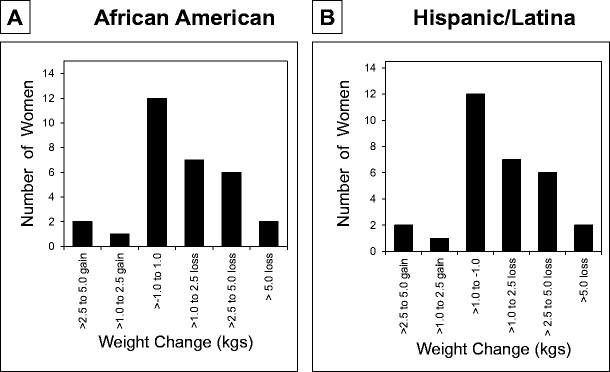
Frequency distribution of weight change in 32 African-American women and 30 Hispanic/Latina women who completed the 12-week community-based Operation Change program

No significant differences were observed in blood pressure or resting heart rates during the 12-week period for the AA or H/L women. Timed 50-ft walking test results improved significantly for the H/L women (*P* < 0.0001) but not for the AA women (*P* = 0.0759). Mean weight decreased significantly at 12 weeks compared with baseline for AA (*P* = 0.04) and H/L (*P* = 0.03) women. However, considerable variability existed for both AA (range 5-kg gain to 11-kg loss) and H/L (range 3-kg gain to 7-kg loss) women (Fig. 2).

### Outcomes

**Fig. 3:**
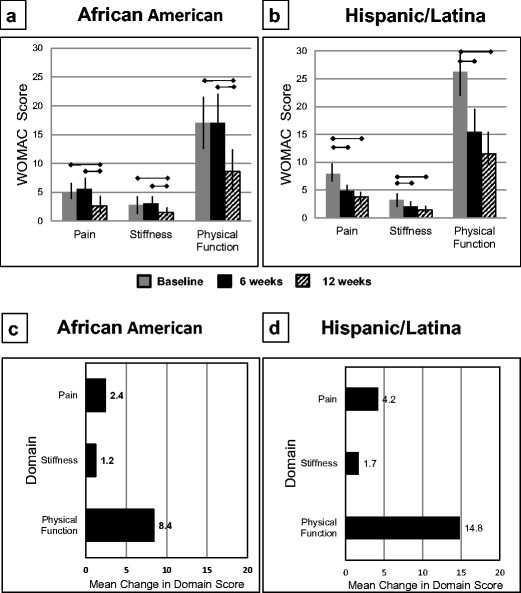
Western Ontario and McMaster Universities Arthritis Index (WOMAC) scores for the **a** 32 African-American women (scores unavailable at 6 weeks for one woman) and **b** 30 Hispanic/Latina women who participated in the Operation Change Program. The results are presented as means and 95% confidence intervals. Asterisk represents statistical significance at *P* < 0.05. The change in each WOMAC domain score is presented for the **c** African-American and **d** Hispanic/Latina women

Patient-reported outcomes were obtained at baseline, 6 weeks, and 12 weeks for all participants except 1 AA participant at 6 weeks. For the WOMAC, significant improvements in pain, stiffness, and physical functioning subscores (all *P* < 0.0001) were observed for both AA and H/L groups (Fig. 3) during the 12-week period. All differences in mean WOMAC scores were greater than the minimal clinically important differences for each subscore [25].

**Fig. 4:**
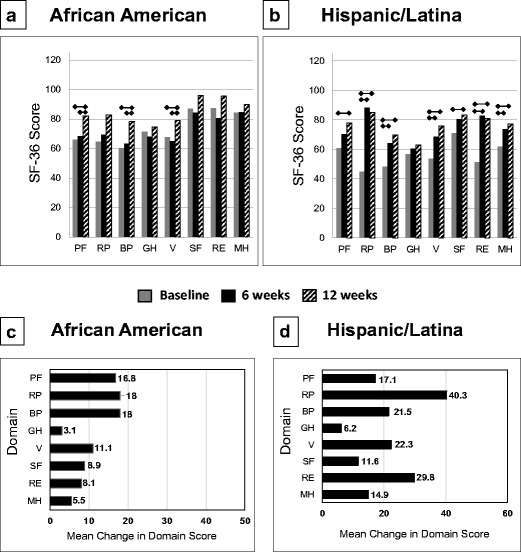
The mean Short Form-36 Health Survey scores for the **a** 32 African-American women (scores unavailable at 6 weeks for one woman) and **b** 30 Hispanic/Latina women who participated in the 12-week Operation Change program. The change in each SF-36 domain score is presented for the **c** African-American and **d** Hispanic/Latina women. The results are presented as means and 95% confidence intervals. Asterisk represents statistical significance at *P* < 0.05. BP bodily pain, GH general health, MH mental health, PF physical functioning, RE role emotional, RP role physical, SF social functioning, V vitality

At 12 weeks, AA women showed significant improvements in SF-36 subscores for physical functioning, bodily pain, and vitality (all *P* < 0.01). Improvements were also observed for role-physical, social functioning, and role-emotional, but these changes were not significant (Fig. 4a). There was no notable change in the general health subscore for the AA participants. H/L women showed significant improvement for all SF-36 subscores by 12 weeks (Fig. 4b), with the exception of general health (*P* = 0.298). All differences in mean SF-36 scores were greater than the minimal clinically important differences, except for social functioning [25, 26].

For the AA women, there was an association between compliance (number of sessions attended) and the SF-36 BP subsection (*r* = 0.379, *P* < 0.05) and with the SF-36 Vitality subsection (*r* = 0.357, *P* < 0.05). Although there were no statistically significant associations for the H/L women, two subsections approached significance: WOMAC-stiffness (*r* = 0.334, *P* = 0.071) and SF-36 physical function (*r* = 0.355, *P* = 0.054).

**Fig. 5:**
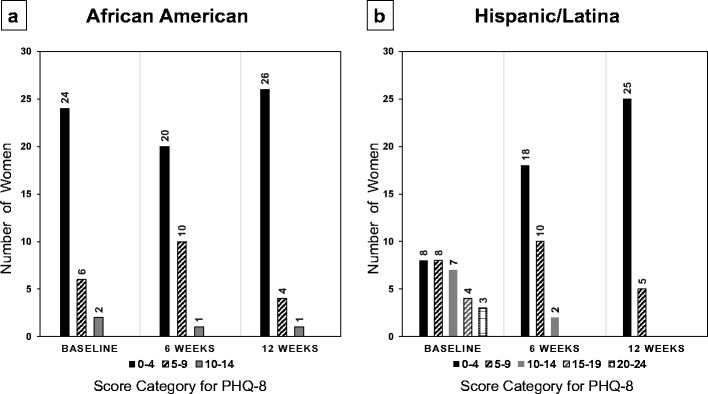
For the 8-Item Patient Health Questionnaire (PHQ-8), the frequency of each score category of **a** 32 African-American women (scores unavailable at 6 weeks for one woman) and **b** 30 Hispanic/Latina women who participated in the 12-week Operation Change program. The PHQ-8 uses the following score categories: 0–4 not significant, 5–9 mild, 10–14 moderate, 15–19 moderately severe, and 20–24 severe

AA women’s PHQ-8 scores were lower at 12 weeks than at baseline, although the difference was not significant (*P* = 0.077) (Fig. 5a). Many AA participants (53%) were in the range indicating no significant depression symptoms (score 0–4) for all three time periods. H/L women showed a significant decrease in PHQ-8 scores between baseline and 6 weeks and between baseline and 12 weeks (*P* < 0.0001) (Fig. 5b). Whereas only 27% of H/L women scored in the “no significant depressive symptoms” range at baseline, 57% scored at this level at 6 and 12 weeks.

#### Motivational Interviewing

All participants ranked as high the importance of goal setting, their readiness to set goals, and their confidence in their ability to do so. On a 10-point visual analog scale, the mean values of each component exceeded 9 points at 6 and 12 weeks.

## Discussion

The Operation Change program was introduced to middle-aged and elderly AA or H/L women in two underserved communities in the South Side of Chicago and led to measurable improvements in outcomes. Using validated instruments, we observed decreases in pain and stiffness, improved physical functioning, and diminished depression in AA and H/L women with early-stage arthritis and obesity, as well as a corresponding significant but modest decrease in weight for some participants during the 12-week study period. A significant decrease in the frequency of depressive symptoms was observed, although this cannot be correlated independently with the physical activity component of the program.

Regular, moderate-intensity physical activity has been reported to decrease the severity of arthritis and its associated pain, fatigue, and stiffness [27]. Little information has been published concerning exercise among AA or H/L women with OA. Wyatt et al. [28] reported that 117 AA participants (93% women; 56% obese) who completed the Arthritis Foundation’s 6-week Walk with Ease program experienced significant decreases in pain, fatigue, and stiffness, which were maintained at 1-year follow-up. Parker et al. [29] found that pain intensity and mood scores improved significantly in AA, Hispanic, and non-Hispanic white participants with walking. The 112 participants were primarily women (83%), and although BMI and obesity status were not reported, 32% of participants were diabetic. The results of these studies agree with the experiences of the AA and H/L participants in Operation Change.

Physical activity trials indicate that 9 to 87% (mean 45%) of participants do not adhere to an intervention [30] and that high dropout rates impede maintenance of physical activity programs for populations similar to those in this study [31, 32]. Barriers include time limitations due to work-life obligations, physical and mental fatigue, depression, negative attitudes toward exercise, including fear of exercise (apprehension regarding pain or falling), and lack of role models [8, 31, 33–35]. The physical environment may present challenges because of poor safety (poor sidewalk or road conditions, inadequate or nonexistent street lights, lack of personal safety) and lack of access to healthy foods (i.e., food deserts) and the corresponding availability of unhealthy foods [12, 31, 36]. Some Operation Change participants described lack of support from spouses to lose weight, unwillingness to change eating habits, and unwillingness to exercise in a program with “thin” participants. The biggest challenge may be behavioral change itself (e.g., increasing activity level) [37]. Most adults, regardless of sex, race, or ethnicity, do not exercise, and exercise participation declines with increasing age [38].

Operation Change offers three critical elements: (1) A focus on increasing mobility rather than losing weight may make the goal appear more attainable to participants, (2) the network of support created by Operation Change can encourage success, and (3) motivational interviewing enhances behavior change. It has been used by large musculoskeletal programs [34, 39] and has been shown to help patients with fibromyalgia [27], diabetes [40], and chronic heart failure [41] to increase their physical activity by helping them overcome ambivalence about changing their behavior [27, 34, 39]. In group-based interventions, physical activity can be coupled with counseling, which includes discussion of self-regulatory skills to help establish behavioral change [42]. Operation Change is a paradigm shift from more traditional programs.

There is the possibility of the Hawthorne effect with any patient-reported outcomes assessment [43]. For example, some participants may report better results to keep the program going, whereas other participants may report worse results, at least initially, because they want to show that their situation is “worse” than that of other participants. Participants were referred to or signed up for the study on the basis of their health status or interest in improving. Although they knew they were being observed, they understood that this observation was being conducted to improve their health and that of others like them. This understanding showed in the results of the study. Participants were asked to complete evaluations of the study at three time points. There was consistency between the two measures of quality of life—the WOMAC and the SF-36. The program evaluation asked both quantitative and qualitative participant perceptions of the programs and ways to improve it. It was learned, by using these evaluations, that participants enjoyed the program tremendously and wanted it to continue.

Strengths of the program include that it was community-based (i.e., designed to be implemented and operationalized with community resources and by community members, who may or may not be associated with healthcare agencies) and that discussion topics reflected the interests of participants. The staff were of the same race or ethnicity as the participants; culturally or racially concordant care has been shown to be more satisfying and to result in positive patient outcomes [44]. A multi-strategy approach was used by Operation Change to encourage behavioral change: a variety of activities, education by physicians and trained healthcare providers, and motivational interviewing. The program overcame some of the obstacles of the built environment by providing a safe space for group physical activities.

A major challenge for the program was the shortage of highly educated professionals who work within the AA and H/L communities. In the H/L group, use of a translator was found to hinder participant engagement; we recommend having bilingual presenters. A limitation of this pilot study was that there were no control groups. This decision was made, after consultation with the community, to avoid possible treatment contamination after community participants raised concerns about the perception of unfairness (i.e., that the treatment group would be receiving “more” than the control group) and the likelihood of communication between treatment and control groups. The AA and HL groups were significantly different in age, although this occurred by chance within the established inclusion criteria. Another limitation is that there was no long-term evaluation of individual sustainability of increased activity level of this 12-week program. Other limitations are the relatively small sample size and the inclusion of some participants with knee pain but lack of physician-reported knee OA.

This study supports the feasibility and efficacy of a community-based program for AA and H/L women that encourages them to take an active role in living with arthritis and incorporates shared decision-making, cultural sensitivity, and a focus on movement and activity. Future studies should address the long-term sustainability of behavioral change regarding physical activity levels.
